# Machine Learning-Based Analysis of Magnetic Resonance Radiomics for the Classification of Gliosarcoma and Glioblastoma

**DOI:** 10.3389/fonc.2021.699789

**Published:** 2021-08-20

**Authors:** Zenghui Qian, Lingling Zhang, Jie Hu, Shuguang Chen, Hongyan Chen, Huicong Shen, Fei Zheng, Yuying Zang, Xuzhu Chen

**Affiliations:** ^1^ Department of Neurosurgery, Beijing Tiantan Hospital, Capital Medical University, Beijing, China; ^2^ Department of Radiology, Beijing Tiantan Hospital, Capital Medical University, Beijing, China; ^3^ School of Mathematical Sciences, Nankai University, Tianjin, China

**Keywords:** gliosarcoma, glioblastoma, machine learning, radiomics, differentiation

## Abstract

**Objective:**

To identify optimal machine-learning methods for the radiomics-based differentiation of gliosarcoma (GSM) from glioblastoma (GBM).

**Materials and Methods:**

This retrospective study analyzed cerebral magnetic resonance imaging (MRI) data of 83 patients with pathologically diagnosed GSM (58 men, 25 women; mean age, 50.5 ± 12.9 years; range, 16-77 years) and 100 patients with GBM (58 men, 42 women; mean age, 53.4 ± 14.1 years; range, 12-77 years) and divided them into a training and validation set randomly. Radiomics features were extracted from the tumor mass and peritumoral edema. Three feature selection and classification methods were evaluated in terms of their performance in distinguishing GSM and GBM: the least absolute shrinkage and selection operator (LASSO), Relief, and Random Forest (RF); and adaboost classifier (Ada), support vector machine (SVM), and RF; respectively. The area under the receiver operating characteristic curve (AUC) and accuracy (ACC) of each method were analyzed.

**Results:**

Based on tumor mass features, the selection method LASSO + classifier SVM was found to feature the highest AUC (0.85) and ACC (0.77) in the validation set, followed by Relief + RF (AUC = 0.84, ACC = 0.72) and LASSO + RF (AUC = 0.82, ACC = 0.75). Based on peritumoral edema features, Relief + SVM was found to have the highest AUC (0.78) and ACC (0.73) in the validation set. Regardless of the method, tumor mass features significantly outperformed peritumoral edema features in the differentiation of GSM from GBM (*P* < 0.05). Furthermore, the sensitivity, specificity, and accuracy of the best radiomics model were superior to those obtained by the neuroradiologists.

**Conclusion:**

Our radiomics study identified the selection method LASSO combined with the classifier SVM as the optimal method for differentiating GSM from GBM based on tumor mass features.

## Introduction

Gliosarcoma (GSM), a variant of glioblastoma (GBM), differs from GBM in many respects (1). GSM is associated with lower ratios of epidermal growth factor receptor (EGFR) and O^6^-methylguanine-DNA methyltransferase (MGMT) promoter methylation without isocitrate dehydrogenase (IDH) mutations as well as the expression of the v-raf murine sarcoma viral oncogene homolog B1(BRAF) gene at codon 600 (BRAF V600E) (2–6). Clinically, GSM is associated with a higher ratio of extracranial metastasis (7, 8) and a poorer prognosis (3, 9–11). These molecular, genetic, and clinical differences between GSM and GBM indicate that the former may be treated as a unique entity.

While the similarity in the clinical presentation of the two types of tumors underscores the importance of their radiological differentiation, most of the radiological signs of the two tumors overlap (2, 4). Prior imaging research has therefore sought to find a method by which to reliably distinguish the two types of tumors: peritumoral edema seen on routine magnetic resonance imaging (MRI) is more severe in patients with GSM (1, 2), and other imaging modalities, including diffusion weighted imaging (DWI), perfusion weighted imaging (PWI), and magnetic resonance spectroscopy (MRS), have also proven to be helpful in the identification of the tumors (7, 12). However, these imaging methods have not been substantive enough to guide clinical practice due to some limitations. First, qualitative radiological features are susceptible to intra and interobserver variability and lacking reproducibility among evaluators. Second, these radiological modalities only focus on the tumor masses of GSM and GBM when peritumoral edema also requires attention.

Radiomics, a new method for imaging data analysis, has been successfully used for the differentiation of central nervous system tumors: e.g., differentiation between primary central nervous system lymphoma and atypical GBM (13), between GBM and metastasis (14–16), and between GBM and anaplastic oligodendroglioma (17). Like any high-throughput data-mining field, the curse of dimensionality presents a challenge for radiomics analysis. Feature selection is the process of removing irrelevant features that are most conducive to reducing the difficulty of learning task and minimizing the risk of overfitting. This study extracted a large panel of radiomic features from the tumor masses and peritumoral edema of GSM and GBM to inform an optimal machine learning-based algorithm for differentiating GSM from GBM.

## Materials And Methods

### Patient Enrollment

The ethics committee of our hospital approved this retrospective study. This study enrolled 83 patients with GSM (58 men, 25 women; mean age, 50.5 ± 12.9 years; range, 16-77 years) between July 2009 and August 2018 and 100 consecutive patients with GBM (58 men; 42 women; mean age, 53.4 ± 14.1 years; range, 12-77 years) between December 2016 and February 2017. The inclusion criteria for this study were as follows: (I) pathologically confirmed GBM or GSM, as defined by the World Health Organization (WHO) criteria; (II) available preoperative multi-parametric MRI data, including T2-weighted imaging (T2WI) and contrast enhanced (CE) data; (III) patients with no history of preoperative treatment for the tumor before receiving MR; and (IV) available clinical data. Patients were excluded if (I) preoperative MR images were not available in our institute; (II) the images were inadequate for image analysis (for example, they featured obvious artifacts); (III) the lesion showed no enhancement on post-contrast images; or (IV) the lesion was recurrent or had received previous treatment. The clinical and imaging characteristics of all patients were retrospectively assessed, including age, gender, tumor location, and the identification of intra-tumoral necrosis and cystic changes and peritumoral edema. The flowchart of 83 patients with GSM and 100 patients with GBM is presented as **Supplementary Figure 1**. The patients were randomly assigned to either the training (n = 93) or validation groups (n = 90).

### MRI Data Acquisition and Region of Interest Segmentation

MRI data included pre- and post-contrast scanning. The detailed scanning parameters are shown in **Supplementary Table 1**. The presence of intra-tumoral necrosis and cystic changes and peritumoral edema were determined for each case. The intra-tumoral necrosis and cystic changes were defined as low signal intensity without enhancement on post-contrast images and high signal on T2WI. The peritumoral edema was defined as low signal intensity around enhanced tumors and high signal on T2WI. The identification of intra-tumoral necrosis, cystic changes, and peritumoral edema were performed by two of the co-authors; conflicting opinions were resolved with discussion.

Several postprocessing steps following the acquisition of MR images were performed to reduce data heterogeneity bias. The adjustment of image resolution was first conducted to resample all voxel size to 3.00 × 3.00 × 3.00 mm^3^ without gaps between consecutive slices for each MRI image. Image intensity normalization transformed MR imaging intensity into standardized ranges (0–1). The contour of the tumor on axial images in the CE sequence and the high signal around the tumor in the T2 sequence (the tumor itself and peritumoral edema) were manually segmented into region of interest (ROI) on multiple slices with the opensource software MRIcro (http://www.mccauslandcenter.sc.edu/mricro/). The ROI of the peritumoral edema on CE images was generated by the voxel-wise subtraction of the contrast enhancement in CE sequence from high signals on T2WI using FSL (http://fsl.fmrib.ox.ac.uk/fsl/fslwiki/FSL).

### Radiomic Feature Extraction and Stability Evaluation

PyRadiomics (http://readthedocs.org/projects/pyradiomics/) computed a total of nine feature categories, including first-order statistics, shape descriptors, texture classes (gray level co-occurrence matrix, GLCM), gray level run length matrix (GLRLM), and gray level size zone matrix (GLSZM), and six built-in filters (wavelet, Laplacian of Gaussian (LoG), square, square root, logarithm, and exponential), resulting in a total of 1,303 radiomic features (13 shape features, 18 first-order intensity statistics features, 68 texture features, 86 square features, 86 square root features, 86 logarithm features, 86 exponential features, 172 LoG features, and 688 wavelet features). First-order features are intensity-based statistical features describing the distribution of voxel intensities. Shape features describe the size and shape of the ROIs. GLCM, GLRLM and GLSZM features are all texture-related features defined by different computations based on the gray level of the image. All of the features were defined in compliance with the Imaging Biomarker Standardization Initiative (IBSI). All the radiomics features were listed in the **Supplementary Table 2**.

### Feature Selection and Classification

A total of three feature selection methods based on statistical approaches were applied in this study: least absolute shrinkage and selection operator (LASSO), Relief and Random Forest (RF). While LASSO and RF are embedded methods, Relief is a filter method. The embedded methods (LASSO and RF) and filter method (Relief) are commonly and effectively used feature selection methods. From the performance of the final model, the wrapped feature selection is better than the filtered feature selection, but the model needs to be trained multiple times, so the computational cost is relatively large. We chose these methods mainly because of their efficiency and popularity among previous studies. In the LASSO algorithm, the shrinkage parameter lambda was identified when the misclassification error was smallest in 10-fold cross-validation. The LASSO, Relief, and RF curve analysis were conducted based on the “glmnet”, “vsurf”, and “CORElearn” packages by R software (version 3.4.0, R Foundation for Statistical Computing), respectively. Then, three machine-learning classifiers were then applied for feature classification: adaboost classifier (Ada), support vector machine (SVM), and RF. These classifiers are widely used pattern recognition tools and imported from the Python (version 3.6.4) machine learning library named scikit-learn (version 19.0).

### Differentiation Performance of the Radiomics Models

The three subsets of selected features were then used as an input to each of the three machine-learning classifiers, which generated nine (3×3 = 9) radiomics models. We applied 5-fold cross-validation as the criteria for each of the nine radiomics models in the training cohort. The differentiation performance was evaluated in the validation cohort. The area under the curve (AUC) and accuracy (ACC) from the receiver operating characteristic curve analysis were calculated to evaluate the differentiation performances of the radiomics models. The optimal thresholds of the AUCs were determined by maximizing the sum of the sensitivity and specificity values calculated for the differentiation of GBM from GSM.

**Figure 1:**
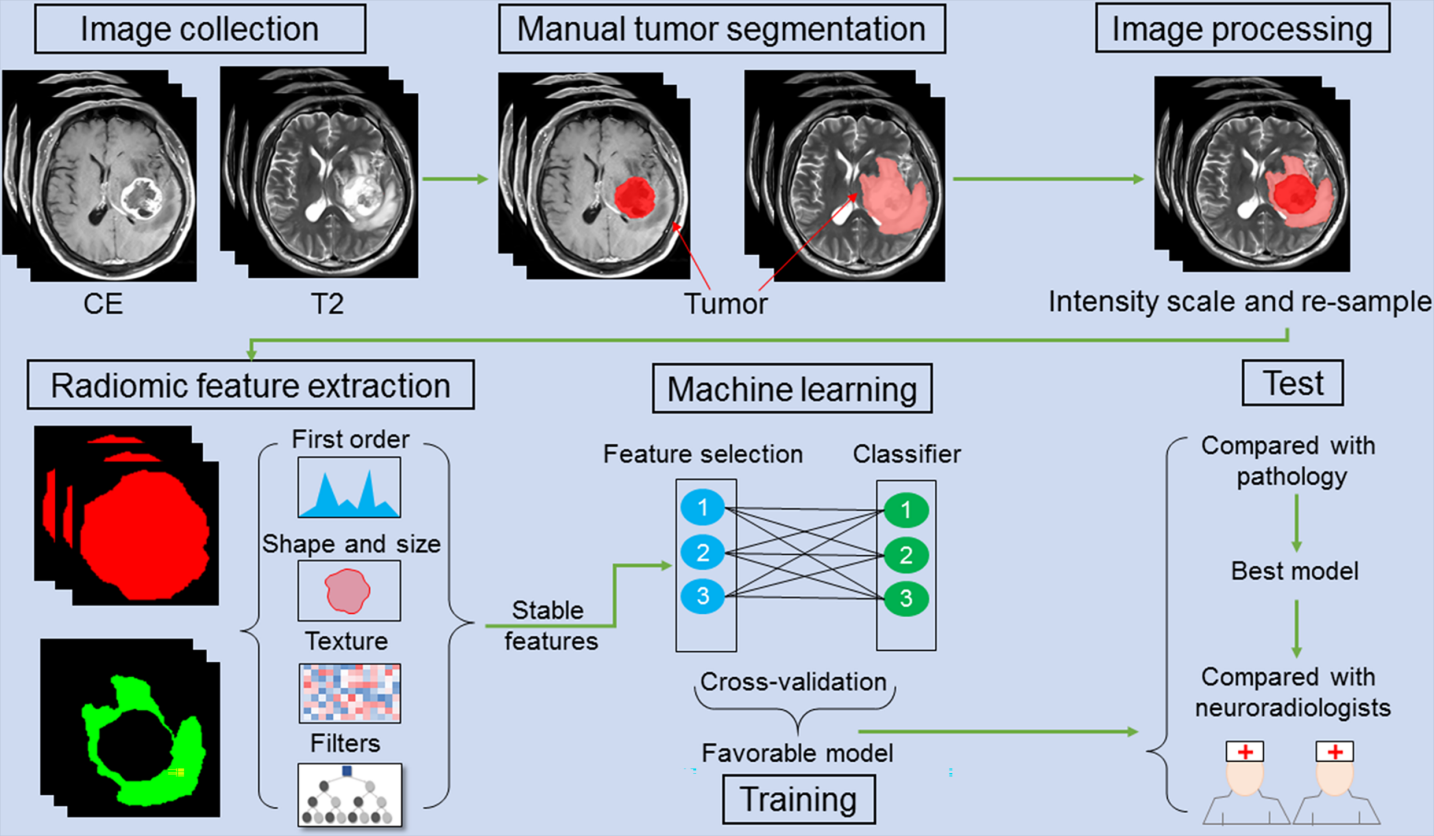
A schematic figure shows the radiomic analysis process. After feature extraction, stable features are selected. Three feature selection and classification methods are combined with favorable models selected and cross-validated in the training cohort. In an independent validation cohort, the optimal model is identified by comparing with pathology. The performance of the optimal model is compared with that of the two neuroradiologists.

To compare the differentiation performances of the radiomics models and neuroradiologists in differentiating GBM from GSM, we employed the two aforementioned neuroradiologists, who were blinded to the clinical and pathological data, to manually differentiate the GBM from GSM according to all of the sequences (T1WI, T2WI, and CET1WI) showing on the Picture Archiving and Communication Systems (PACS), just as the daily radiological diagnosis workflow before ROI segmentation. They were allowed to see the full MRI images used in this study for the first time. The results of inter-observer variation and concordance with final histopathology statistics between the two neuroradiologists are shown in **Supplementary Table 3**. The chi-square test was performed to compare the proportion of predicted GBM/GSM between the neuroradiologists and the best radiomics model. The entire analysis process is shown in **Figure 1**.

### Statistical Analysis

Differences in the clinical and MRI characteristics between GBM and GSM were evaluated using the *t*-test and chi-square test. *P-*values of less than 0.05 were considered to indicate statistical significance. The statistical analysis and figure plots were performed using R (version 3.0.1; http://www.R-project.org) and SPSS (SPSS Inc.).

## Results

### Clinical and Routine MRI Characteristics

GBM and GSM showed no difference in patient age and gender (P=0.151; χ^2^ = 2.758, P=0.097). The ratio of intra-tumoral necrosis and cystic changes was 98.8% (82/83) and 95.0% (95/100) among patients with GSM and GBM, respectively. This difference was non-significant (**Table 1**). The prevalence of peritumoral edema was 94.0% (78/83) and 83.0% (83/100) among patients with GSM and GBM, respectively. The difference was significant (χ^2^ = 5.166, *P*=0.023).

**Table 1:** Clinical and MRI characteristics of patients with GSM and GBM.

	Training cohort			Validaion cohort		
	GSM (n=43)	GBM(n=50)	*P* value	GSM (n=40)	GBM(n=50)	*P* value
Age (years)	51.1	51.6	0.884^†^	49.8	55.2	0.044^†^
Sex						
Female	9	23	0.011^*^	16	19	0.847^*^
Male	34	27		24	31	
Localization						
Supratentorial	43	47	0.296^*^	39	50	0.444^*^
Infratentorial	0	3		1	0	
Necrosis						
Yes	42	47	0.720^*^	40	48	0.501^*^
No	1	3		0	2	
Edema						
Yes	39	42	0.337^*^	39	41	0.047^*^
No	4	8		1	9	

^*^Chi-square test, ^†^Student’s t-test. GBM, glioblastoma; GSM, gliosarcoma; MRI, magnetic resonance imaging.

### Selection of Stable Features

We calculated intraclass correlation coefficient (ICC) to select for the robustness of radiomic features in tumor mass and peritumoral edema. For the tumor mass, 918 of the 1,303 (70.5%) extracted radiomic features showed high stability, including 13 shape features, 18 first-order intensity statistics features, 70 texture features, 84 square features, 81 square root features, 80 logarithm features, 89 exponential features, 179 LoG features, and 304 wavelet features. For the peritumoral edema, 815 of the 1,303 (62.5%) extracted radiomic features showed high stability, including 13 shape features, 18 first-order intensity statistics features, 64 texture features, 70 square features, 89 square root features, 65 logarithm features, 80 exponential features, 162 LoG features, and 254 wavelet features.

**Figure 2:**
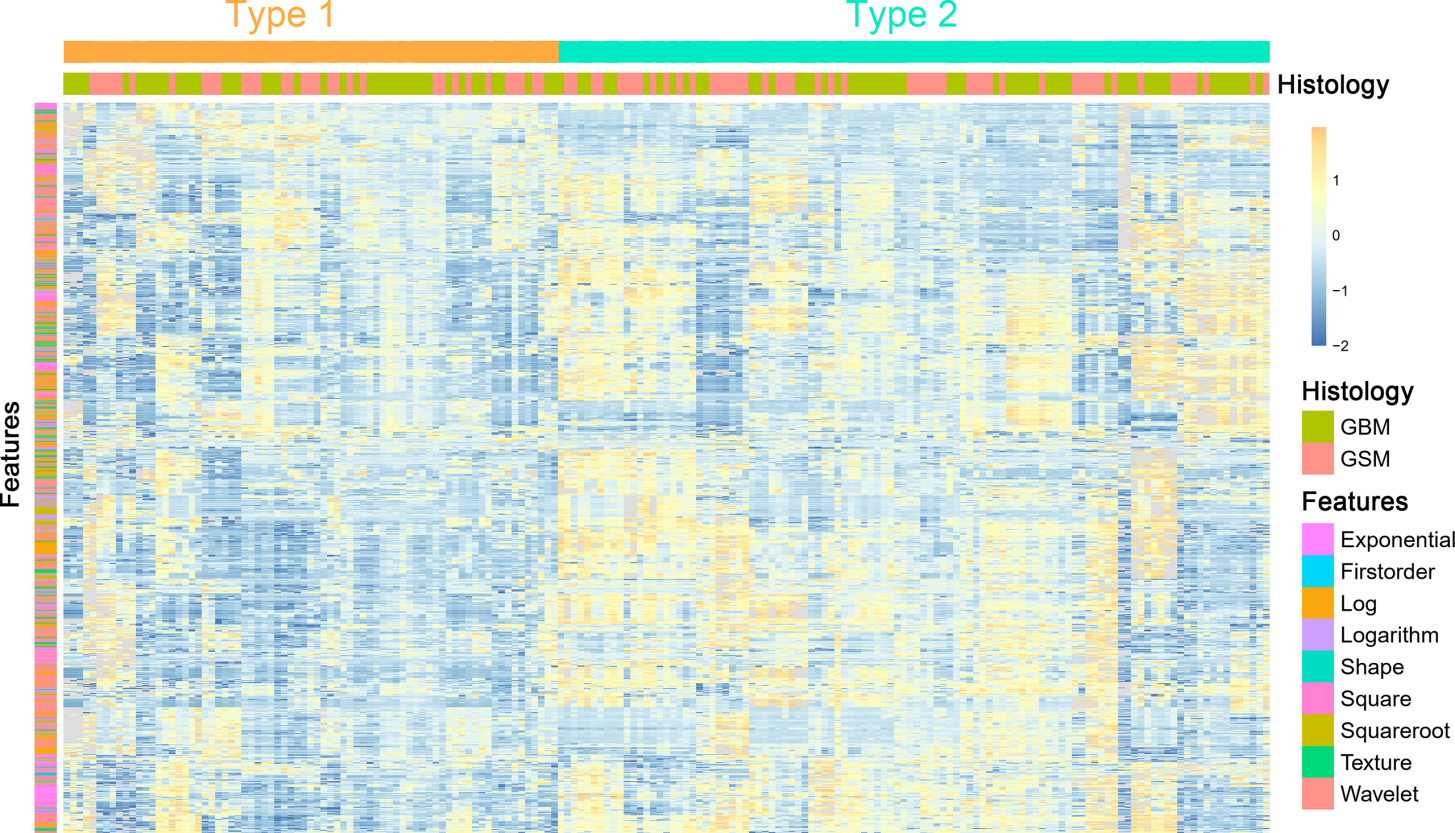
A heat map shows the stable radiomic features. Each column and row correspond to one patient and z-score normalized radiomic feature, respectively.

Unsupervised clustering of these stable features was conducted and presented as a heat map to yield two imaging subtypes (**Figure 2**). However, the association between the imaging and histology subtypes was not obvious.

### Feature Selection and Radiomics Model Construction

**Figure 3:**
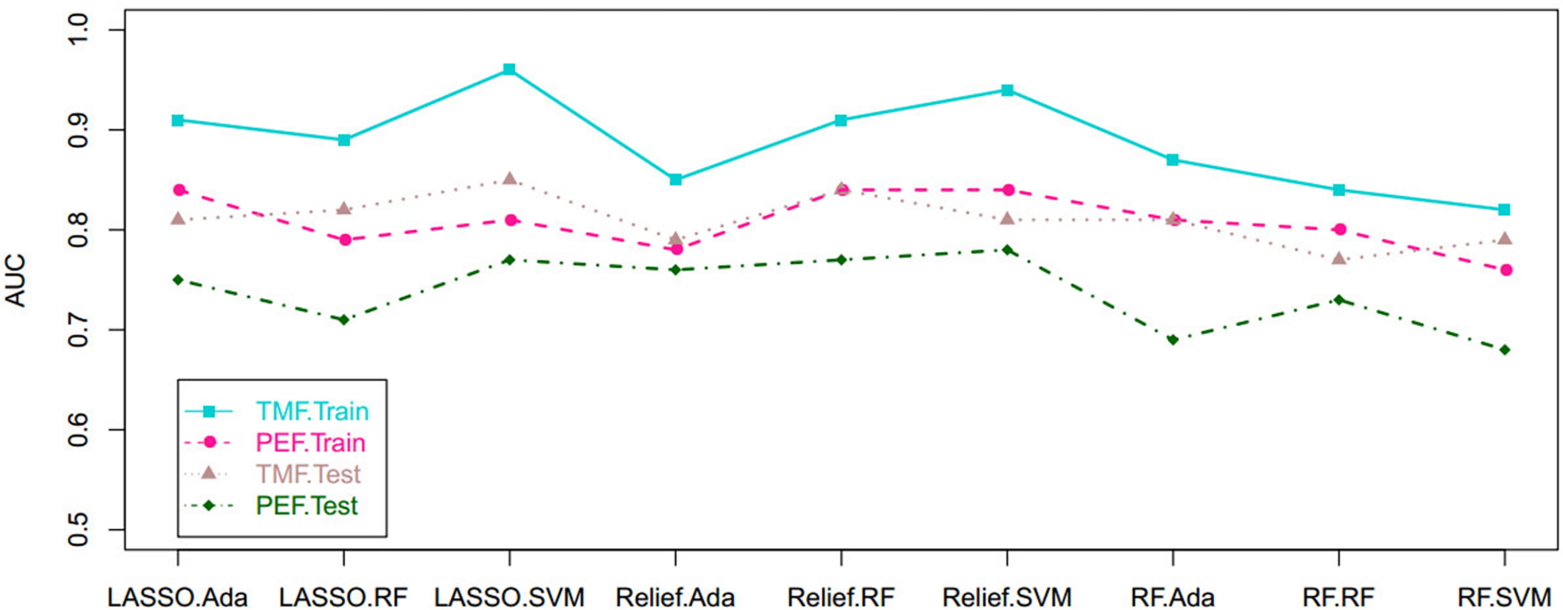
Scatterplots depict the AUC of the cross-combination methods based on the features derived from the tumor and peritumoral edema, respectively. AUC, area under the curve.

**Figure 4:**
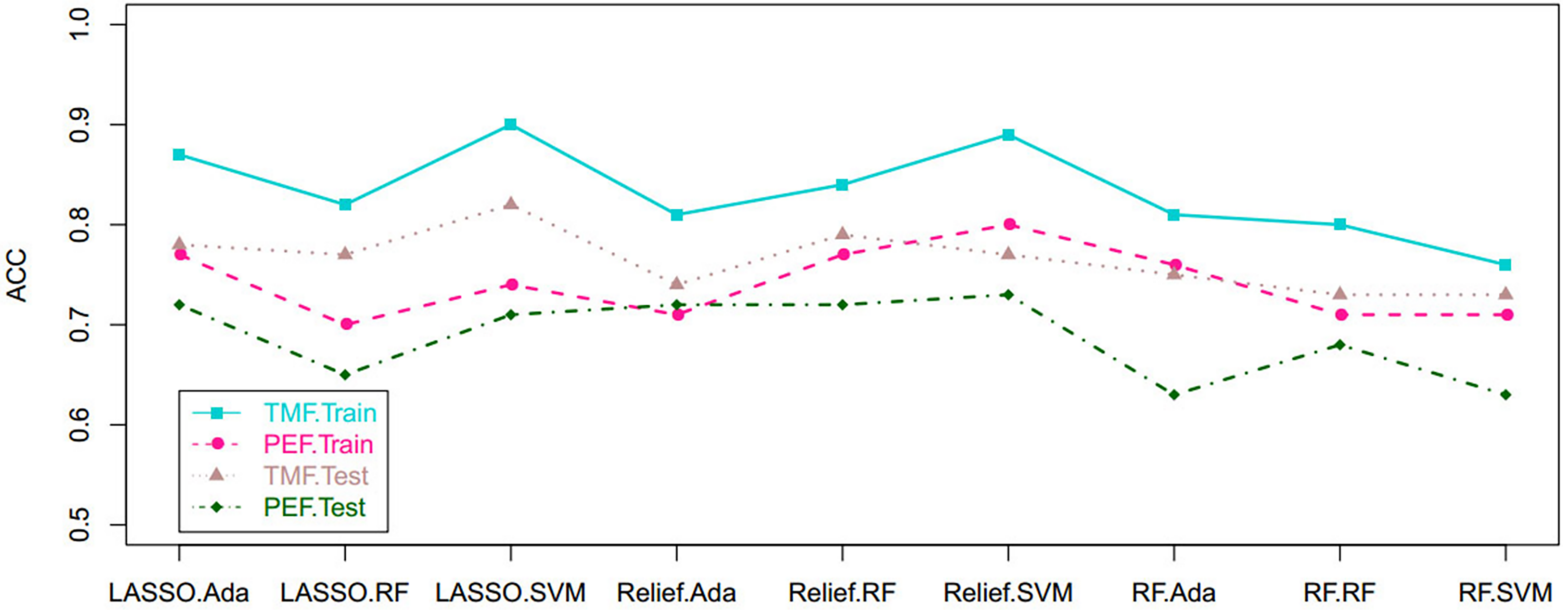
Scatterplots show the ACC of the cross-combination methods based on the features derived from the tumor and peritumoral edema, respectively. ACC, accuracy.

**Figure 5:**
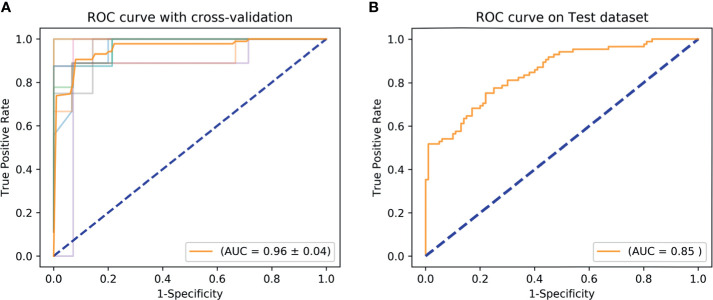
ROC curve shows the optimal classifier for differentiating GSM from GBM. **(A)** The AUC of 5-fold cross-validated ROC is 0.96 in the training set. **(B)** The AUC of 5-fold cross-validated ROC is 0.85 in the validation set. AUC, area under the curve; GBM, glioblastoma; GSM, gliosarcoma; ROC, receiver operating characteristic.

Based on tumor mass features in the training set, the selection method LASSO + classifier SVM was found to feature the highest AUC (0.96) and ACC (0.85), followed by those of Relief + RF (AUC = 0.94, ACC = 0.81), LASSO + RF (AUC = 0.91, ACC = 0.84), and LASSO + Ada (AUC = 0.91, ACC = 0.81; **Tables 2** **–** **4** and **Figures 3**, **4**). A similar result was found using the tumor mass features in the validation set: the selection method LASSO + classifier SVM featured the highest AUC (0.85) and ACC (0.77), followed by those of Relief + RF (AUC = 0.84, ACC = 0.72) and LASSO + RF (AUC = 0.82, ACC = 0.75). In both the training and validation set, regardless of the method, tumor mass features significantly outperformed those of the peritumoral edema in the differentiation of GSM from GBM (*P*< 0.05). The illustration of the 5-fold cross-validated ROC curve of the LASSO + SVM radiomics model in the training cohort and ROC curve of the LASSO + SVM radiomics model in the validation set are shown in **Figure 5**.

**Table 2:** The AUC of the cross-combination methods.

AUC	Ada	RF	SVM
TMF			
LASSO	0.91 (0.81)	0.89 (0.82)	0.96 (0.85)
Relief	0.85 (0.79)	0.91 (0.84)	0.94 (0.81)
RF	0.87 (0.81)	0.84 (0.77)	0.82 (0.79)
PEF			
LASSO	0.84 (0.75)	0.79 (0.71)	0.81 (0.77)
Relief	0.78 (0.76)	0.84 (0.77)	0.84 (0.78)
RF	0.81 (0.69)	0.80 (0.73)	0.76 (0.68)

The AUC of the cross-combination methods based on tumor mass and peritumoral edema features is showed in the training set (no brackets) and the validation set (in brackets). Ada, adaboost; AUC, area under the receiver-operating characteristic curve; LASSO, least absolute shrinkage and selection operator; PEF, peritumoral edema feature; RF, random forest; SVM, support vector machine; TMF, tumor mass feature.

**Table 3:** The ACC of the cross-combination methods.

ACC	Ada	RF	SVM
TMF			
LASSO	0.83(0.74)	0.81(0.75)	0.87(0.77)
Relief	0.77(0.70)	0.80(0.72)	0.84(0.75)
RF	0.77(0.71)	0.76(0.70)	0.71(0.65)
PEF			
LASSO	0.73(0.68)	0.69(0.63)	0.71(0.67)
Relief	0.72(0.64)	0.75(0.70)	0.79(0.73)
RF	0.74(0.63)	0.71(0.68)	0.71(0.63)

The ACC of the cross-combination methods based on tumor mass and peritumoral edema features are showed in the training set (no brackets) and the validation set (in brackets). ACC, accuracy; ACC, accuracy; Ada, adaboost; LASSO, least absolute shrinkage and selection operator; PEF, peritumoral edema feature; RF, random forest; SVM, support vector machine; TMF, tumor mass feature.

**Table 4:** Comparison of predictive performance between radiomic model and neuroradiologists in the validation set.

	Sensitivity, *P*	Specificity, *P*	Accuracy, *P*
Neuroradiologist with 3 years of experiences	0.40, <0.001^*^	0.44, <0.001^*^	0.42, <0.001^*^
Neuroradiologist with 10 years of experiences	0.70, 0.015^*^	0.34, <0.001^*^	0.50, <0.001^*^
LASSO_SVM	0.78, —	0.76, —	0.77, —

^*^Chi-square test. LASSO, least absolute shrinkage and selection operator; SVM, support vector machine.

To avoid biases and confirm the efficacy of the radiomics model, we compared the performance of the selection method LASSO + classifier SVM in 90 validation cases with that of experienced and inexperienced raters. As shown in **Table 4**, the clinical performance of the LASSO + SVM radiomics model was superior to that of the neuroradiologists in terms of sensitivity, specificity, and accuracy.

## Discussion

This retrospective study developed and validated a favorable predictive model with radiomics features extracted from tumor mass and peritumoral edema to distinguish GSM from GBM. Importantly, the trend of the diagnostic performance of this machine-learning radiomics model was similar in the training set, validation set, and cross-validation analysis. In our study, two neuroradiologists independently rendered diagnosis of the two kinds of tumors based on the routine MRI; their accuracy was less than 50.0%, lower than the accuracy of the radiomics analysis, suggesting the superiority of radiomics relative to human analysis in distinguishing GSM from GBM.

In agreement with previous research (18, 19), our study indicated that GSM usually showed enhancement on the solid component with peritumoral edema on routine MRI. These findings, however, are insufficient to inform the distinction of GSM from GBM. Some advanced imaging modalities, such as DWI, PWI, and MRS (7, 12, 20), have therefore been used to better identify the characteristics of GSM. On DWI, the thicker or more solid components of GSM show a restricted diffusion ratio of as high as 72.7% (8/11) (7); on PWI, the tumor featured high perfusion (7); on MRS, GSM shows a lactate peak indicating local necrosis and hypoxia of the tumor and a higher lipid-choline ratio than do GBM (12, 20). These indices obtained from the advanced MR modalities were all derived from analysis of the solid part of the tumor. However, due to the fact that GSM and GBM usually evince necrosis and cystic changes, a comprehensive differentiation between the two tumors should simultaneously involve the solid part and non-solid components. The peritumoral region, which usually shows as edema, is also neglected during differentiation.

In our study, the differentiation between GSM and GBM not only included the whole part of the lesion but also the peritumoral edema outside of the lesion. Our investigation revealed that, based on the peritumoral edema region, the two tumors can be differentiated with the radiomics method of Relief + SVM (AUC, 0.78; ACC, 0.73). Showing as high signal intensity on T2WI, this region included both vasogenic edema and the infiltration of tumor cells (21–23). However, compared with this region, analysis of the tumor mass itself allowed for the more efficient differentiation between tumor types. This can be explained by the fact that there are far more tumor cells in the region of tumor mass than in the peritumoral region. Moreover, the whole region of the tumor mass, including necrosis, cystic changes, and other non-enhanced components, was analyzed for its capacity to inform differentiation. As previous studies that employed PWI, DWI, and MRS (7, 12), only focused on the solid part of the two kinds of tumors, our analysis is more factual and practicable.

Radiomics is an emerging non-invasive method that extracts high-dimensional sets of imaging features to build appropriate models for survival prediction (24), distant metastasis prediction (25), and molecular characteristics classification (26). However, dimensionality is a critical challenge in radiomics analysis and limits the potential of the radiomics model. Hence, this study compared three feature selection methods and classification methods for improving the stability and classification performance of the radiomics model. After performing nine cross-combinations comparisons, we found the LASSO selection method and the classifier SVM to best differentiate of GSM from GBM. The LASSO is a regularization technique used to minimize the number of non-zero elements and make the solution unique (27). It is therefore often used to solve the problem of large sets of radiomics features derived from a relatively small sample size. The SVM is a powerful classification algorithm that can estimate the classification probabilities and control complexity. These properties account for its effective application in the fields of neuroimaging and molecular biology (16, 28) and its superb pairing with the LASSO selection method in our radiomics analysis.

Our study has several limitations. First, it may be subjective to selective bias as a retrospective study. Second, the scanning parameters were not uniform, requiring the preprocessing of the data. Third, compared with the large radiomic features dataset, the sample size was relatively small. Therefore, our results may be caused by overfitting. Fourth, only T2WI and axial post contrast T1WI were used in our radiomic analysis, multi-model imaging data (such as DWI, PWI, MRS) needs to be integrated into our model in the future, to improve its performance. Finally, being a single center study, our study is lack of external independent validation.

In conclusion, this retrospective study presents the machine learning-based MR radiomics model as a non-invasive tool for preoperatively differentiating GSM from GBM with favorable predictive accuracy and stability. Prospective studies are needed to further validate its classification ability.

## Data Availability Statement

The raw data supporting the conclusions of this article will be made available by the authors, without undue reservation.

## Ethics Statement

The studies involving human participants were reviewed and approved by Beijing Tiantan Hospital. Written informed consent for participation was not provided by the participants’ legal guardians/next of kin because: As a retrospective study, it was approved by our institute committee without the informed consent of the patients.

## Author Contributions

ZQ and LZ performed study design, information collection, statistical analysis, and manuscript editing. HC, HS, and XC guided study design, reviewed images, and revised manuscript. JH and SC provided technical support. FZ and YZ collected images and clinical information. All authors contributed to the article and approved the submitted version.

## Funding

This work is supported by the National Natural Science Foundation of China under grant numbers 81772005 and 82001897 and Collaborative innovative major special project supported by Beijing Municipal Science & Technology Commission under grant number Z191100006619088.

## Conflict of Interest

The authors declare that the research was conducted in the absence of any commercial or financial relationships that could be construed as a potential conflict of interest.

## Publisher’s Note

All claims expressed in this article are solely those of the authors and do not necessarily represent those of their affiliated organizations, or those of the publisher, the editors and the reviewers. Any product that may be evaluated in this article, or claim that may be made by its manufacturer, is not guaranteed or endorsed by the publisher.
