# Corrigendum: Machine Learning-Based Analysis of Magnetic Resonance Radiomics for the Classification of Gliosarcoma and Glioblastoma

**DOI:** 10.3389/fonc.2021.774369

**Published:** 2021-10-18

**Authors:** Zenghui Qian, Lingling Zhang, Jie Hu, Shuguang Chen, Hongyan Chen, Huicong Shen, Fei Zheng, Yuying Zang, Xuzhu Chen

**Affiliations:** ^1^ Department of Neurosurgery, Beijing Tiantan Hospital, Capital Medical University, Beijing, China; ^2^ Department of Radiology, Beijing Tiantan Hospital, Capital Medical University, Beijing, China; ^3^ School of Mathematical Sciences, Nankai University, Tianjin, China

**Keywords:** gliosarcoma, glioblastoma, machine learning, radiomics, differentiation

In the published article, there was a mistake in the order of authors. The co-first author was incorrectly written as the second author. The correct author list with the correct author order appears below.

“Zenghui Qian^1†^, Lingling Zhang^2†^, Jie Hu^1^, Shuguang Chen^3^, Hongyan Chen^2^, Huicong Shen^2^, Fei Zheng^2^, Yuying Zang^2^, Xuzhu Chen^2*^”

The authors apologize for this error and state that this does not change the scientific conclusions of the article in any way. The original article has been updated.

## Publisher’s Note

All claims expressed in this article are solely those of the authors and do not necessarily represent those of their affiliated organizations, or those of the publisher, the editors and the reviewers. Any product that may be evaluated in this article, or claim that may be made by its manufacturer, is not guaranteed or endorsed by the publisher.

