# Supplementary material for: Machine Learning-Based Analysis of Magnetic Resonance Radiomics for the Classification of Gliosarcoma and Glioblastoma
Source: Front Oncol. 2021 Aug 20;11:699789. doi: 10.3389/fonc.2021.699789 (PMC8417735; doi:10.3389/fonc.2021.699789)
Supplement: Supplementary file 1 [file Table_1.docx]

**SUPPLEMENTARY MATERIALS**

**S1: The inclusion and exclusion criteria of cohort**

1. ***The inclusion and exclusion criteria of gliosarcoma***

Pathological database search between July 1, 2009 and August 31, 2018 using the term “gliosarcoma”, n=173

Recurrent, n=14

Without preoperative MRI in our institute, n=29

Preoperative MRI without post-contrast images, with obvious artifact, n=6

Underwent specific treatment (radiotherapy, et al) aimed at the tumor before MRI scanning, n=41

83 consecutive patients

**Supplementary Figure 1a:** The flowchart of the patient cohort with gliosarcoma

1. ***The inclusion and exclusion criteria of glioblastoma***

Pathological database search between December 1, 2016 and February 28, 2017 using the term “glioblastoma”, n=154

Recurrent or secondly operated, n=8

Without preoperative MRI in our institute, n=34

Preoperative MRI without post-contrast images, tumors showing no enhancement on

post-contrast images, with obvious artifact, n=10

Underwent specific treatment (radiotherapy, et al) aimed at the tumor before MRI scanning, n=2

100 consecutive patients

**Supplementary Figure 1b:** The flowchart of the patient cohort with glioblastoma

**S2: The detailed magnetic resonance imaging scanning protocol and parameters**

For the 83 patients with cerebral gliosarcoma (GSM) and 100 patients with glioblastoma (GBM), the scanning protocol included pre-and postcontrast scanning. The pre-contrast scanning included sagittal T1-weighted imaging (T1WI), axial T1WI and axial T2-weighted imaging (T2WI). Once the pre-contrast scanning was finished, the contrast media of dimeglumine gadopentetate was injected with a dose of 0.2ml/kg via the antecubital vein of the patient. After the injection, the scanning continued with axial, sagittal and coronal T1WI images obtained. The magnetic resonance (MR) scanners are shown in the following Supplementary Table 1.

**Supplementary Table 1:** The MR scanner types in patients with GSM and GBM

| MR scanners | T2WI | | | |
| --- | --- | --- | --- | --- |
|  | TR(ms) | TE(ms) | Slice/Gap (mm) | Matrix |
| GE Discovery MR750 (n=35) | 10221-10696 | 84 | 5.00/6.00 | 512×512 |
| GE Medical System Genesis Signa (n=32) | 4900 | 116-117 | 5.00/6.00 | 512×512 |
| Siemens MAGNETOM TrioTim System (n=69) | 4500 | 84 | 5.00/6.00 | 384×324 |
| Siemens MAGNETOM Verio (n=31) | 6000 | 97 | 5.00/6.00 | 640×640 |
| GE Signa HDe (n=16) | 2700 | 103 | 5.00/6.00 | 512×512 |
|  | Post-contrast axial T1WI | | | |
| GE Discovery MR750 (n=35) | 2804 | 19 | 5.00/6.00 | 512×512 |
| GE Medical System Genesis Signa (n=32) | 2031 | 19 | 5.00/6.00 | 512×512 |
| Siemens MAGNETOM TrioTim System (n=69) | 2000 | 9.8 | 5.00/6.00 | 512×432 |
| Siemens MAGNETOM Verio (n=31) | 1900 | 9.4 | 5.00/6.00 | 512×496 |
| GE Signa HDe (n=16) | 2378 | 10 | 5.00/6.00 | 512×512 |

GBM = glioblastoma, GSM = gliosarcoma, MR = magnetic resonance, TE = time of echo, TR = time of repeatation, T1WI = T1-weighted imaging, T2WI = T2-weighted imaging
